# A Holistic Landscape Description Reveals That Landscape Configuration Changes More over Time than Composition: Implications for Landscape Ecology Studies

**DOI:** 10.1371/journal.pone.0150111

**Published:** 2016-03-09

**Authors:** Anne Mimet, Vincent Pellissier, Thomas Houet, Romain Julliard, Laurent Simon

**Affiliations:** 1 UMR 7533 CNRS-Paris 1-Paris 7-Paris 8-Paris 10, Laboratory of Social Dynamics and Space Recomposition [LADYSS], 2 rue Valette, FR-75005, Paris, France; 2 UMR 7204 MNHN-CNRS-UPCM, Center of Conservation Science [CESCO], 55 rue Buffon, FR-75005, Paris, France; 3 UMR 5602 CNRS-UTM, Geography of the Environment [GEODE], 5 Allée A. Machado, FR-31058, Toulouse, Cedex 1, France; University of Sydney, AUSTRALIA

## Abstract

**Background:**

Space-for-time substitution—that is, the assumption that spatial variations of a system can explain and predict the effect of temporal variations—is widely used in ecology. However, it is questionable whether it can validly be used to explain changes in biodiversity over time in response to land-cover changes.

**Hypothesis:**

Here, we hypothesize that different temporal *vs* spatial trajectories of landscape composition and configuration may limit space-for-time substitution in landscape ecology. Land-cover conversion changes not just the surface areas given over to particular types of land cover, but also affects isolation, patch size and heterogeneity. This means that a small change in land cover over time may have only minor repercussions on landscape composition but potentially major consequences for landscape configuration.

**Methods:**

Using land-cover maps of the Paris region for 1982 and 2003, we made a holistic description of the landscape disentangling landscape composition from configuration. After controlling for spatial variations, we analyzed and compared the amplitudes of changes in landscape composition and configuration over time.

**Results:**

For comparable spatial variations, landscape configuration varied more than twice as much as composition over time. Temporal changes in composition and configuration were not always spatially matched.

**Significance:**

The fact that landscape composition and configuration do not vary equally in space and time calls into question the use of space-for-time substitution in landscape ecology studies. The instability of landscapes over time appears to be attributable to configurational changes in the main. This may go some way to explaining why the landscape variables that account for changes over time in biodiversity are not the same ones that account for the spatial distribution of biodiversity.

## Introduction

One of the main goals of landscape ecology is to provide a better understanding of how populations and communities respond to changes in land use and cover [LUC] in space and time [1]. Ecological conservation requires both an understanding of the ecological processes associated with landscape composition and configuration, and a complete understanding of the landscape dynamics of composition and configuration induced by LUC changes which strongly impact biodiversity. In this study, a LUC patch is an isolated tract of homogeneous land use cover (e.g. an agricultural plot). Landscape composition is determined by the type and proportion of LUC patches present in elementary landscape units [2,3]. Landscape configuration is determined by the diversity and spatial arrangement of LUC patches in landscape units determined by various landscape variables such as distance between patches or patch size [4]. The influence of landscape composition and configuration on biodiversity has been widely investigated in the literature based on two strong assumptions: the habitat/matrix paradigm [5,6] and the space-for-time substitution (also termed the ergodic principle or static approach) [7] applied to landscape ecology studies to predict future biodiversity dynamics [8]. In the habitat/matrix paradigm, which is derived from island biogeography theory and metapopulation theory [9,10], habitat patches are viewed as islands surrounded by an inhospitable matrix. The space-for-time substitution states that the average of the value of a single system over time is equal to the average of the values of *n* identical systems at any given moment. In our context, this would mean that the consequences for biodiversity of changes in landscape composition and configuration over time could be evaluated by studying the spatial variations of biodiversity with respect to spatial variations of landscape composition and configuration. Obtaining time series data for land cover and biodiversity data over periods long enough to show significant change in biodiversity and landscape involves substantial economic costs and is time consuming. The saving that can be made with space-for-time substitution is probably the main reason it being so widely used in landscape ecology [11].

Several authors have shown that composition is usually more important than configuration when explaining spatial biodiversity patterns [3,12,13]. Using the space-for-time substitution, the results of those studies are widely extrapolated to conclude that composition changes are likely to be much more important than configuration changes in explaining species and community changes. Thus it is claimed that composition is the element to focus on to protect biodiversity [14].

These views have been challenged. The habitat/matrix paradigm has been shown to lead to an oversimplification of landscape processes [3,5]. Studies using holistic descriptions of the landscape, i.e. detailing the composition and configuration of the various types of land cover, have revealed that small variations in configuration can significantly alter the suitability of a landscape for a species, even when compositions are comparable, i.e. there are similar amounts of the various types of land cover in the landscape [12,15,16]. Long-term studies are recognized to be preferable to space-for-time substitution in studying biodiversity dynamics [17]. Some studies have challenged the space-for-time approach in landscape ecology by showing that the landscape variables that are used to explain the spatial distribution of biodiversity are not necessarily the same variables as are used to explain biodiversity dynamics over time [18,19]. Other studies have indicated that the spatial and temporal landscape variables explaining the current distribution of biodiversity are not necessarily the same [20–24]. One explanation for these observations may be that when studying biodiversity dynamics, the space-for-time substitution assumes that historical and current landscapes do not differ significantly across sites [7], which is probably wrong or at least not verified in most studies [25].

Given these challenges, it is critical to understand the dynamics of landscape composition versus configuration over time in response to subtle land cover changes in order to improve the combination of conservation concerns and human activities in landscapes [26–31].

Composition and configuration variables are strongly correlated and interrelated [32–34]. The decline in extent of any particular LUC type affects both landscape composition and configuration. For instance, the conversion of a small fraction of a patch of farmland into urbanized land will modify the proportions of the two types of land cover in the landscape. As only a small area is directly involved, the proportions of the two types of land cover will change only slightly, and the conversion will induce only minor changes in overall landscape composition. However, this conversion will also affect the landscape configuration by modifying the distance between patches (isolation), as well as their size, shape and/or number. It will also modify heterogeneity by changing the relative proportions of the two types of land cover, and one type may simply disappear from the landscape. Thus, this change may have greater repercussions for landscape configuration than composition, as it will probably have consequences on more metrics and for both types of land cover involved (isolation, size, and shape).

Using time series of a holistic description of the landscape in the Seine-et-Marne department near Paris, France, the present study tests the validity of the space-for-time substitution on the landscape scale to assess landscape composition and configuration changes over time. It focuses on understanding the repercussions of subtle changes in LUC over time on landscape composition and configuration, which are assumed to be key landscape characteristics for biodiversity.

## Materials and Methods

### Study site and land cover data

**Fig 1:**
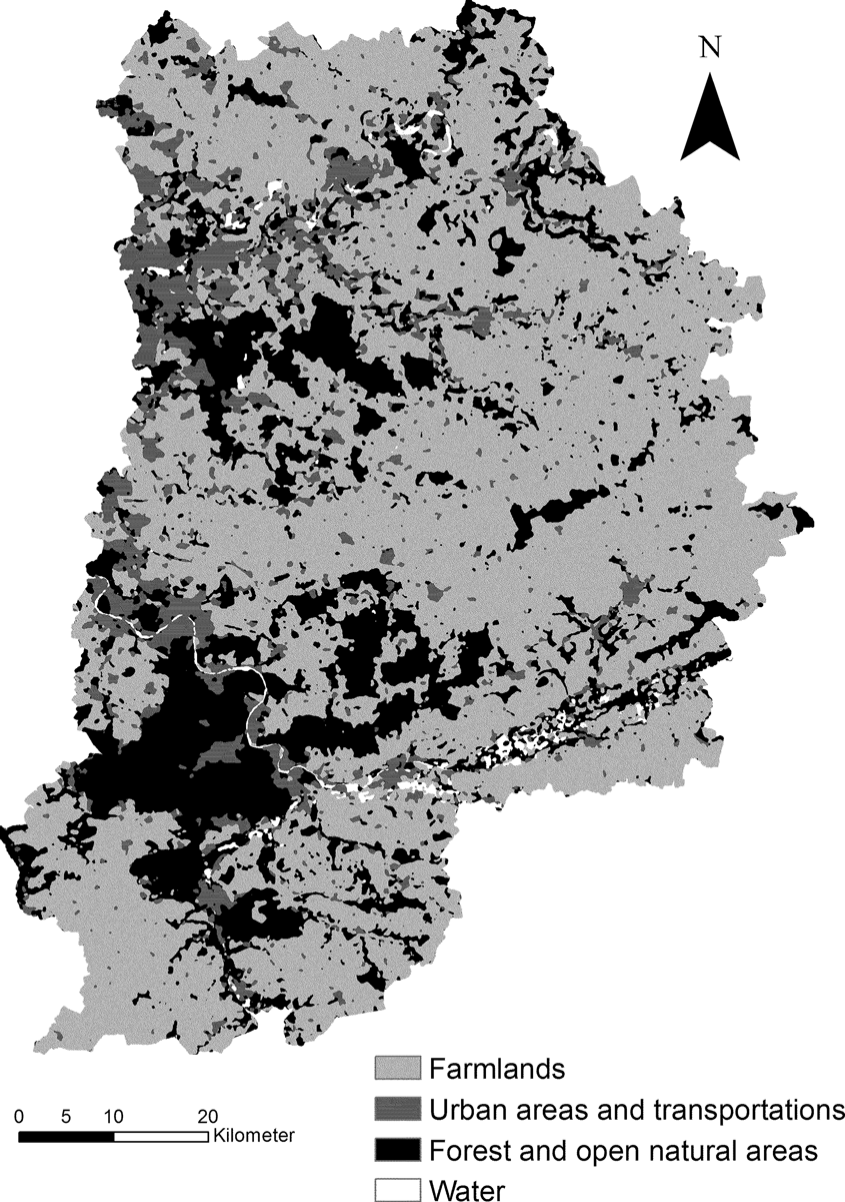
Main LUC types in the Seine-et-Marne department in 2003.

The study was conducted in Seine-et-Marne, which is the largest administrative department around Paris, covering 5915 km^2^ (48°06’– 49°7’ N; 2°23’– 3°32’ W). In 2007, the population of the region was nearly 1 353 946, with a density of 218 inhabitants per km². Farmland is the dominant LUC type, with more than 60% of the territory being allocated to farming. The proximity of the department to Paris has resulted in substantial LUC changes, dominated by urbanization, the development of transport networks, and loss of farmland and forest. Natural areas of interest remain in some locations across the department, mostly in the form of forested areas and wetlands (Fig 1).

The Île-de-France Institute of Urban Planning and Development (*IAU*) provided the Land Use Pattern (LUP) database, which is a LUC database covering the Île-de-France region for 1982 and 2003 at 25 m resolution with 1:5000 geometric precision. These data were derived from the interpretation of aerial photographs and additional data on municipalities’ territories, building cartography and type provided by the municipalities. The information provided by the LUP database was simplified by grouping its 83 LUC divisions into just six LUC classes:

- Agricultural areas: areas given over to arable farming and pasture.
- Urbanized areas: built areas, urban parks and gardens, building land, or swimming pools.
- Forest: natural woodlands, forests, and poplar stands.
- Water and wetlands: rivers, other bodies of water, and wetlands.
- Transport areas: road, rail, car parks
- Natural open areas: wasteland and cut forest.

### Description of the landscape

The methods and data used to describe the landscapes in the present study have been presented at length in previous studies [15,16] but are briefly described below (for an explanation of the importance of a holistic landscape description in ecology studies see S1 File).

The holistic landscape description was based on two parallel descriptions of landscape units, one for composition and one for configuration. These two descriptions followed the same methodology: landscape metrics were computed and the two sets of metrics reduced by two separate multivariate analyses. This step eliminated co-variation *within* composition and configuration metrics respectively, because landscape metrics are usually closely correlated [32]. This step therefore ensured that the analyses did not include any redundant information. However, the methodology did not prevent covariation *between* composition and configuration as the metrics were obtained from two separate multivariate analyses. This was necessary, because the information common to composition and configuration variables could not be attributed to one or the other of these landscape characteristics and consequently had to be saved in both instances [13].

The region was divided into 6524 hexagonal landscape units (hexagonal cells) with centroids spaced 1 km apart (approximately 78 ha each), roughly equivalent to the area covered by a circle with a 500 m radius. Because landscape scaling is species-specific [35], we selected a scale that would allow for unique yet comparable landscape descriptions. This scale was selected on the basis of the literature as suitable for multi-species studies of bird-landscape relationships for forest species [36] and farmland species [13,37]. Each of these units was then considered to represent a landscape. Landscape composition and configuration were computed for each landscape and for the two years of the LUC data. The landscape variables of the two years of the study (1982 and 2003) were integrated in the multivariate analyses.

Landscape composition was assessed by computing the respective proportions of the six LUC classes in each landscape unit and analyzing them through correspondence analysis [CA] with the ade4 package [38] for R.2.10.1 [39] [40]. The first three axes explained 86% of the observed variance (Table A in S1 Table). The first axis corresponded to a gradient of human conversion of natural areas, mainly forests, for agricultural purposes (hereafter referred to as the “forest-to-farmland” variable). The second axis corresponded to a gradient of urbanization (hereafter, the “urbanization” variable). Note that historically urbanization in the department is located mainly in the great valleys of the rivers Seine and Marne, which explains the weight of the water/wetlands variable on the second axis. The third axis identified natural wetlands and open natural areas (hereafter referred to as the “decline in natural wetlands” variable).

Rather than being based on the configuration of each LUC type, the landscape configuration was described in terms of the discrimination between dominant LUC classes (over 20% of the area in a hexagonal unit) and minority LUC classes in the landscape unit (under 20% of the landscape unit, see [16], for the rationale for the 20% threshold). Accordingly, landscape metrics were computed for each LUC type and then averaged by dominant (one or two LUC types depending on the landscape units) and minority (zero to five LUC types) LUC types in each landscape unit. This method not only limited the number and complexity of initial configuration variables but also described the landscape configuration without reference to LUC type (and consequently to landscape composition), because the configuration variables referred to the dominant/minority status. By this method, landscape configuration was rendered qualitatively independent of landscape composition: a configuration described by variables of comparable values could exist for different compositions. Thus, an example of a configuration metric is the shape complexity of the patches of the dominant LUC, and not the shape complexity of forest patches. It also allowed for statistical independence between composition and configuration [15,41]. When considering a landscape unit, the information yielded by composition made it possible to identify the dominant and minority LUCs referred to by the configuration variables. Landscape configuration metrics were selected according to the literature to inform the main components of landscape characterization [32,42]. We computed the mean patch perimeter/area ratio, the Euclidean distance between patches, the number of patches, and the mean patch perimeter. Unlike the methodology presented in 14 and 15, we omitted heterogeneity variables from the configuration analyses in the main methodology, as they are often described as composition variables [3,30] (Table B in S1 Table). However, to ensure consistency with the landscape description presented in those two earlier papers, results incorporating heterogeneity variables into the configuration description are presented in Table C in S1 Table. Each metric was computed for both the dominant and minority LUC classes. All of these variables are linked to biodiversity or ecological processes [6,43,44]. All eight variables were computed in a principal component analysis (PCA) using the ade4 package. Based on the eigenvalue plot, we kept the first seven axes, which explained 96% of the observed variance. The first axis provided general information about the configuration at the landscape level, describing a gradient from patchy landscapes to uniform landscapes (referred to as the “decrease in patchiness” variable). The second axis explained the decreasing isolation of the dominant and minority LUC patches (the “decreasing isolation” variable). The third axis provided information about patch shape complexity (the “decrease in shape complexity” variable). The fourth axis increased with complex shapes and size of the patches of minority LUCs (the “shape complexity and size of minority LUC” variable). The fifth axis separated complex-shaped dominant LUC landscapes from complex-shaped minority LUC landscapes (the “Dominant to minority shape complexity” variable). The sixth axis highlighted landscapes with numerous patches of minority LUC and large distances between patches of dominant LUC (the “Minority patchiness between distant dominant LUC” variable). Finally, the seventh axis highlighted landscapes with a small number of clustered minority LUC patches (the “Clustered minority LUC” variable). The landscape composition and configuration metrics used in the multivariate analyses are available in S2 and S3 Files.

### Comparison of the variations in composition and configuration over time

We followed a five-step method for comparing the variations in composition and configuration over time:

1. **Computation of composition and configuration variables**: We computed the values for the three composition variables and the seven configuration variables extracted from the CA and the PCA for each landscape in 1982 and 2003. After this step, the composition variables have comparable *within* variation and the configuration variables comparable *within* variation.
2. **Standardization between composition and configuration variables**: This is the fundamental step in making composition and configuration comparable in space and time, independently of the number of variables used to build them. We standardized each composition and configuration variable by the total (i.e. summed) variation of the selected variables of the CA and the PCA respectively to (a) obtain comparable values for the composition and configuration variables for 1982 and 2003, and so to [b] ensure we observe only time variations when considering the differences between 1982 and 2003, following the formula:
  $${Stdz\_LandVar}_{ij}={LandVar}_{ij}/\sum_{i=1}^{n}sd({LandVar}_{i})$$ (1)
  with *LandVar*_*i*_ *as* the composition or configuration variable i, j the year and n the number of selected composition or configuration variables of the PCAs.
  Therefore, in each landscape, we divided the values for the composition and configuration variables by the sum of the standard deviations of the selected axes from the multifactorial analysis to which they belonged (i.e. by the total standard deviation of the CA for the three composition variables and by the total standard deviation of the PCA for the seven configuration variables). After standardization, the summed spatial variation of the three composition variables was equal to the summed spatial variation of the seven configuration variables in 1982 and 2003. Thus, standardization gave the same weight to composition and configuration, regardless of the number of variables composing each. Consequently, the amplitudes of variation in composition and configuration (obtained by summing the standard deviations of the composition or configuration variables) were equal in space (and equal to 1), and their changes over time were therefore comparable.
3. **Computation of the standardized changes over time**: We then computed the standardized temporal changes between 1982 and 2003 (*Chg_LandVar*_*i*_) for the three composition and seven configuration variables in each landscape by subtracting the values of these ten variables in 1982 from their values in 2003 by the formula:
  $$Chg\_LandVar_{i}=Stdz_{LandVar}{}_{{}_{i}}(2003(-Stdz_{LandVar}{}_{{}_{i}}\left(1982\right))$$ (2)
  with *Stdz_LandVar* as the standardized composition or configuration variable i in 1982 or 2003.
4. **Total variation of the temporal changes**: We computed the standard deviations (sd) for the standardized temporal changes in each of the composition and configuration variables as a measure of the amplitude of their respective variations over time (*Sd_Chg_LandVar*_*i]*_)_*)*_ by the formula:
  $${Sd\_Chg\_LandVar}_{i}=sd[({Chg\_LandVar}_{i}))$$ (3)
5. **Total amplitude of temporal changes in composition and configuration**: We summed the standardized standard deviations of the temporal changes for the three composition variables and seven configuration variables to obtain the amplitudes of the temporal changes in composition and configuration by the formula:
  $$Tot\_Chg\_Comp=\sum_{i=1}^{n}{Sd\_Chg\_Comp}_{i}$$ (4)
  $$Tot\_Chg\_Conf=\sum_{i=1}^{n}{Sd\_Chg\_Conf}_{i}$$ (5)
  with *Sd_Chg_Comp* the total variation of the temporal change of composition variables i and *Sd_Chg_Conf* the total variation of the temporal change of configuration variables i.
  This step was the equivalent of the sum performed in step (ii) to evaluate global spatial variability and global temporal variability.

To provide a basis for comparison using more conventional metrics we also applied an alternative method to the entire set of landscape metrics (S4 File, Tables A and B in S4 File). This method dispenses with the multivariate analysis step, so it gives the standardized temporal changes of the initial composition and configuration metrics. Although it does not avoid the covariation and standardization issues (as multivariate analyses are not done), the results cannot be used to quantify the differences in temporal variations between composition and configuration.

## Results

Between 1982 and 2003, the net change in LUC concerned 4.6% of the Seine-et-Marne study area, i.e. a mean change of 0.242% per year, whereas the average annual rate of change in France during the period 1990–2000, based on Corine Land Cover data, was estimated to be approximately 0.198% per year [45]. The forest and farmland classes lost large areas, mainly through conversion into open natural and urbanized areas (Tables 1 and 2). Some conversions of urbanized areas to natural open areas and farmland can be observed. They are mainly explained by the conversion of large green areas (such as parks and gardens) that had been for urban use in 1982 into farmland and natural open areas. In many instances these new natural open areas were intended as future construction sites.

**Table 1:** Areas of the six LUC types in 1982 and 2003 in hectares, their respective proportion of the study area as a percentage, and their percentage change with respect to their area in 1982.

a)	1982	2003	Difference between 1982 and 2003	Difference between 1982 and 2003 (% of 1982 area)
**Farmland**	374037 **(63,1%**)	359332 **(60,6%)**	-14705 **(-2,5%)**	**-3,9%**
**Urban**	48769 **(8,2%)**	61953 **(10,5%)**	13184 **(+ 2,2%)**	**+27%**
**Forest**	142876 **(24,1%)**	130523 **(22%)**	-12353 **(-2,1%)**	**-8,6%**
**Open natural**	17162 **(2,9%)**	27906 **(4,7%)**	10744 **(+1,8%)**	**+62,6%**
**Transport**	4092 **(0,7%)**	5749 **(1%)**	1657 **(+0,3%)**	**+40,5%**
**Wetlands/Water**	5863 **(1%)**	7336 **(1,2%)**	**1473 (+0,2%)**	**+25,1%**

**Table 2:** Matrix conversion of LUC changes between 1982 and 2003 in hectares.

	2003					
b]	Farmland	Urban	Forest	Open natural	Transport	Wetlands /Water
**Farmland**	354142	11141	1069	5514	1060	1111
**Urban**	1292	45431	275	1159	356	255
**Forest**	1536	1740	125391	13718	254	236
**Open natural**	2291	3504	3756	7313	73	224
**Transport**	7	48	8	29	3999	1
**Wetlands/Water**	64	89	24	172	6	5508

**Fig 2:**
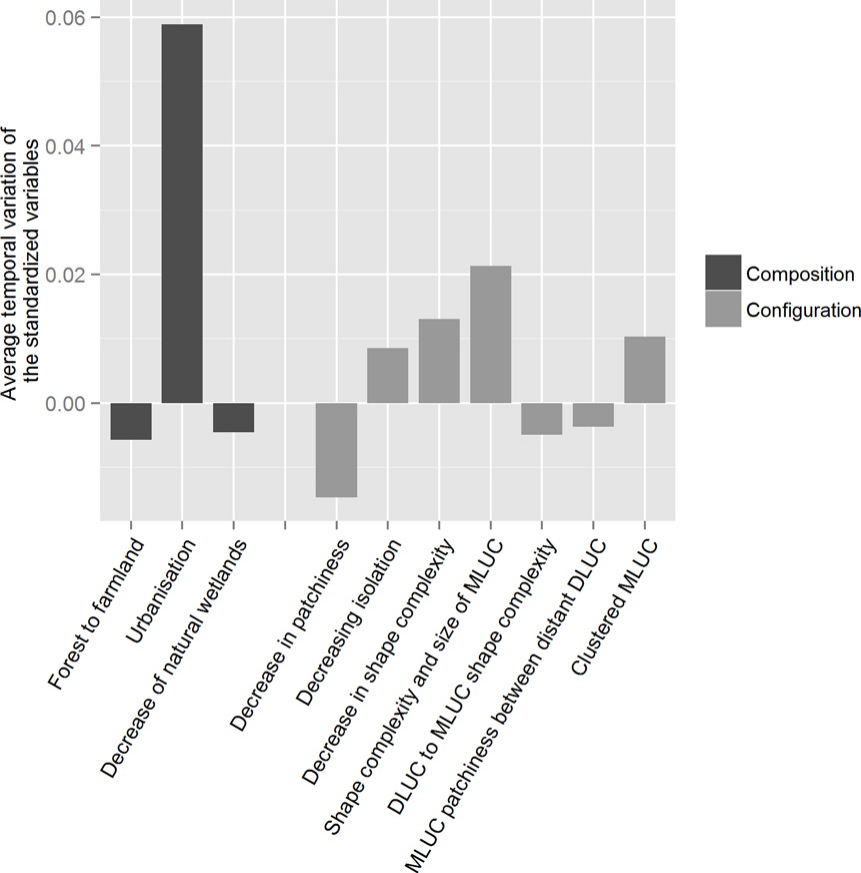
Averaged temporal variation of the standardized three composition and seven configuration variables from the multivariate analyses for the overall landscapes of the study area between 1982 and 2003 (DLUC for dominant LUC, MLUC for minority LUC).

The variable exhibiting the greatest change was a composition variable, “increasing urbanization”, distantly followed by a configuration variable, “Shape complexity and size of minority LUC” (Stdz_LandVar_i_) (Fig 2). The sums of the absolute values of the average changes in composition (0.069) and configuration (0.076) were similar, with configuration changing on average only a little more than composition over time (Fig 2).

**Fig 3:**
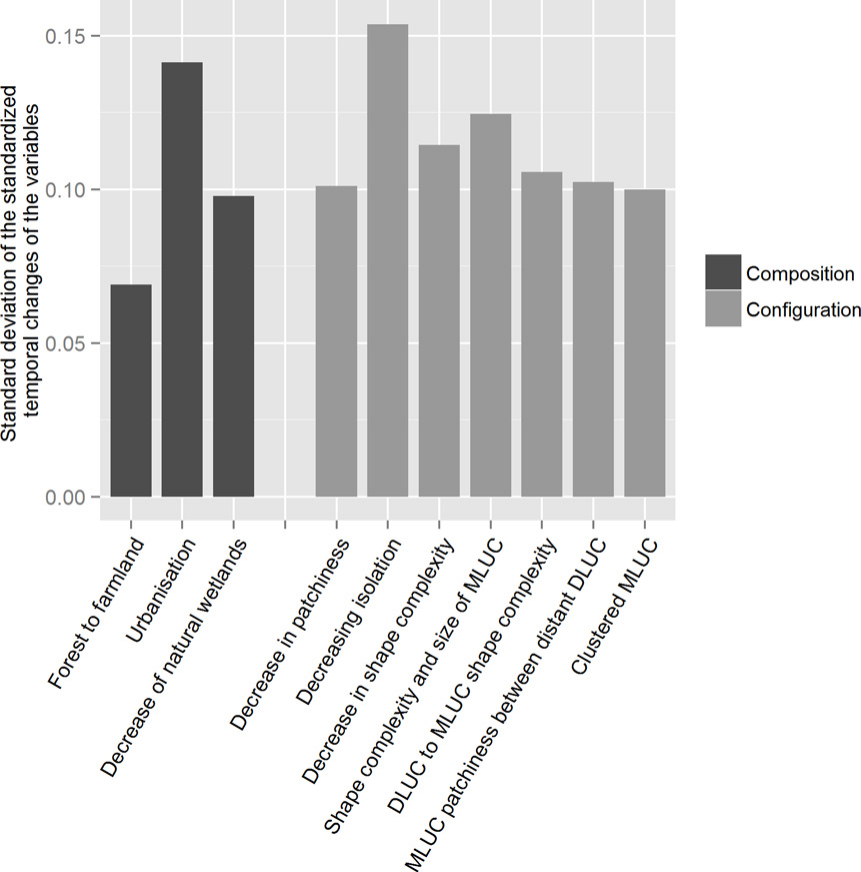
Standard deviations of the standardized temporal changes (Chg_LandVar_i_) for the composition and configuration variables and their sum (DLUC for dominant LUC, MLUC for minority LUC).

The range of the standard temporal changes (Chg_LandVar_i_) was smaller, with values between 0.07 and 0.16 (Fig 3). The variables with the greatest amplitude were again the increasing urbanization (a composition variable) and isolation (a configuration variable), although the latter did not show large changes in averaged temporal variation (Stdz_LandVar_i_) (Fig 3). The other configuration variables had quite comparable standard deviation values, whereas they had different averaged values of change (Fig 3). These results highlight contrasting temporal dynamics of the landscape units for these variables. The sum of the standard deviations of the temporal changes was 0.8 for the configuration (Tot_Chg_Conf) versus 0.31 for the composition (Tot_Chg_Comp) variables (Fig 3). In other words, configuration changes were 2.6 times greater than the composition ones.

The results obtained by the alternative simplified method showed qualitatively similar results, with configuration metrics being on average more variable over time than composition metrics (S1 Fig). The results obtained by the method including heterogeneity variables in the configuration description also yielded similar results but with a reduced difference between the temporal changes of composition and configuration (Figures A and B in S2 Fig), suggesting that heterogeneity is less variable over time than the configuration variables.

**Fig 4:**
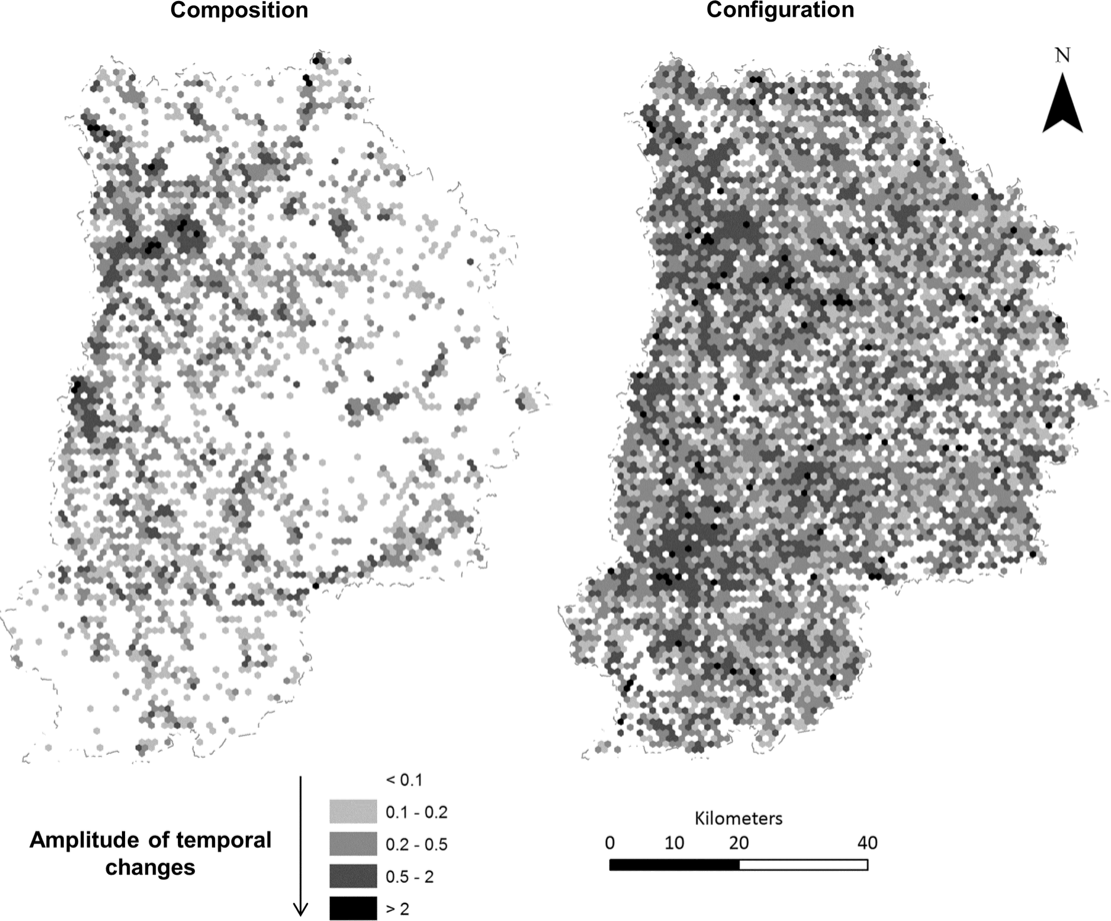
Maps of the changes in the amplitude of the temporal composition and configuration between 1982 and 2003. The amplitudes are computed as the sum of the absolute values of the changes of the standardized composition and configuration variables between 1982 and 2003.

The amplitude maps for the changes in terms of composition and configuration (Fig 4) highlight that most changes were related to configuration. Composition showed greater variation in valleys, and to a lesser extent in urbanized areas and forests, which represent a relatively small proportion of the surface area of the department. Some of the changes in configuration matched composition changes, but configuration changes appeared in other places and were more common in the entire department. They were more marked in forested areas, and were also present and often strong in the intensive agricultural areas of the southwest, northwest and east-central parts of the department. This result reflected two antagonistic landscape dynamics. In urbanized areas, large changes corresponded to conversions of LUC patches of forest, natural open areas and farmland to urban areas, increasing number and the size and of urban patches by aggregation. In forested areas, changes corresponded to increased sprawl and new LUC patches due to forest cutting and management (conversion into natural open areas).

## Discussion/Conclusion

### Configuration varies much more over time than composition: questioning and balancing the response of biodiversity to LUC changes

This study indicates that, under the conditions of our investigation, landscape configuration is more variable over time than landscape composition, up to 2.6 times higher in this study. These results concern the Seine-et-Marne department between 1982 and 2003 and for the given landscape scale. Configuration appears to be more sensitive to LUC changes, suggesting that subtle changes in composition have a marked influence on landscape stability via its configuration. The results highlight the fact that taken independently, the 10 landscape variables (three composition and seven configuration variables) display similar temporal variation. Consequently, the higher temporal variation for configuration than composition is due to the multiple facets of configurational changes induced by land cover changes over time (as a reminder, the variable standardization step made composition and configuration comparable in space and time, independently of the number of variables composing them: we thus expected equal temporal variations between the sums of all composition and configuration variables). For instance, in a simple landscape with two different types of land cover, variations in the total area of one type of land cover will modify the respective amount of the two types of land cover (composition) and have direct consequences for the configuration variables of these two types of land cover (shape, isolation, perimeter, number of patches). In our case, a conversion from one type of land cover to another will affect two composition metrics but probably four configuration metrics (size, distance, perimeter, and/or number of patches).

These results go further than the findings of Long et al. [46] who only considered the changes of a single land cover (forest) in assessing their impacts on forest composition and configuration in landscapes and found that forest composition changed more over time than forest configuration. The differences in results can be explained by the integration of more landscape variables linked to the different LUC types, which better described landscape complexity, i.e. by the use of a holistic approach to landscape description [47]. Moreover these results suggest that different LUC types are not equally involved in composition and configuration changes. The geography (topography, hydrography, etc.) and anthropogenic features (human settlements or transport network, etc.) may influence the trajectories of composition and configuration [30]. In our study, urbanized areas appeared to be highly sensitive to both landscape composition and configuration changes, probably because of the intensity of the LUC changes in these areas. On the other hand, the configuration changes were clearly greater than composition changes in forested and agricultural areas, which are commonly composed of more homogeneous (less fragmented and diversified) landscapes [15].

These emergent properties of LUC changes observed on the landscape scale might alter our understanding of the responses of biodiversity to LUC changes, particularly regarding the spatial causes of changes in biodiversity over time. The landscape variables explaining the spatial distribution patterns of biodiversity are indeed not necessarily the same as those explaining the changes in biodiversity over time [18]. As subtle landscape changes in terms of composition induce larger variations in terms of configuration, the temporal instability of the landscape might increase when considering landscapes using a holistic landscape description. While the stability of a landscape over time is known to be a key driver of biodiversity distribution patterns [48], favouring specialized communities and limiting biological homogenization [21], our results suggest that the use of the space-for-time substitution to estimate the relative importance of composition and configuration changes for biodiversity dynamics may lead to an under-estimation of the importance of configurational changes. This may explain the importance of landscape configuration variables found in previous studies of landscape-biodiversity relationships based on temporal data [20,21] or the weak explanatory power of composition in explaining biodiversity changes [18].

The different speeds of composition and configuration changes should have contrasting impacts on species. Due to their particular preferred habitats and movement abilities, species are not equally dependent on landscape composition and configuration [12]. Therefore, the coincidence of the sensitivity of species to changes in composition and configuration over time could lead to different trajectories of communities in various geographic and anthropogenic contexts. [16] showed that, for the area under study, farmland bird species are more sensitive to variation of configuration than forest species. Therefore, the substantial changes over time in the configuration observed in farmland might have greater consequences for farmland communities than the changes observed in forest areas would have on forest communities. However, an important result here is that landscape instability over time is more largely attributable to configuration than composition changes, when instability is known to be a major factor of biotic homogenization [21,49].

### Limitations and perspectives for landscape ecology and conservation

These results concern only the Seine-et-Marne department over the last 20 years of the twentieth century. Because of the contrasting landscape and human contexts, this department is representative of the main anthropogenic LUC in France and Western Europe. However, similar studies should be conducted in other regions and/or countries to confirm these results. This would help us to better understand and distinguish the spatial and temporal mismatches of landscape composition and configuration changes induced by LUC changes. As landscape patterns can change with scale [1], the importance of landscape extent as well as data resolution should also be tested to define the scales for which these findings are valid.

This study does not focus on the precise consequences for species or biodiversity of identified changes in composition and configuration of landscape. We look only at the amplitude of the repercussions of LUC changes on landscape composition and configuration. Only a holistic approach to landscape, not directed at any particular species or group of species, can be used in addressing this point [47]. This has interesting implications for the way landscape should be described in biodiversity studies. The mechanistic consequences of LUC changes on landscape composition and configuration highlight the fact that their spatial and temporal variations do not have the same amplitudes. Partial description of composition or configuration (i.e. habitat-oriented) would not allow us to observe the same patterns and would limit the captured importance of configuration in explaining both biodiversity distribution and changes over time.

The consequence of this result for biodiversity studies is that extrapolating the relative importance of the composition and configuration to temporal biodiversity changes based only on spatial data is not completely accurate as by definition it ignores how landscape actually changes over time. It questions the validity of the space-for-time substitution in landscape ecology studies, which has been widely applied in ecology and is today called into question [50]. These results indicate only that with the variables widely used to describe landscapes in ecology studies and in a context of disentangling composition from configuration effects, space-for-time substitution should not be considered self-evident and should not be used without further investigation [18].

From a conservation perspective, increased attention should be paid to configuration changes (but overall landscape configuration changes, taking all land cover into account, not only habitat configuration changes in the landscape) which commonly affect landscape stability and appear to be decisive for species distribution, especially in the late stages of fragmentation [51]. More generally, a strong research investment in studying changes in biodiversity over time related to landscape changes is really needed to understand which landscape variables induce change in biodiversity patterns and go beyond the variables explaining the spatial patterns of biodiversity. The recent development of large temporal biodiversity datasets (such as the Breeding Bird Census data developed by numerous countries) and of remote sensing products could be of use in investigating these questions.

## Supporting Information

S1 Table Variables coordinates on axis of (A) the initial composition variables with the three selected axes of the CA of the composition, (B) the initial configuration variables with the seven selected axes of the PCA of the configuration (C) the initial configuration variables with the seven selected axes of the PCA of the configuration when configuration variables include heterogeneity variables.
(DOCX)

S1 File Ecological processes, spatial patterns and landscape descriptions. (DOCX)

S2 File Description of the data set. (DOCX)

S3 File Data set. (XLSX)

S4 File Simplified method applied to the initial set of landscape metrics. (DOCX)

S1 Fig Results of the simplified method. (DOCX)

S2 Fig Results of the analyses including the heterogeneity variables in the PCA of landscape configuration. (DOCX)
