# Supplementary material for: A Holistic Landscape Description Reveals That Landscape Configuration Changes More over Time than Composition: Implications for Landscape Ecology Studies
Source: PLoS One. 2016 Mar 9;11(3):e0150111. doi: 10.1371/journal.pone.0150111 (PMC4784918; doi:10.1371/journal.pone.0150111)
Supplement: S1 Table — Variables coordinates on axis of (A) the initial composition variables with the three selected axes of the CA of the composition, (B) the initial configuration variables with the seven selected axes of the PCA of the configuration (C) the initial configuration variables with the seven selected axes of the PCA of the configuration when configuration variables include heterogeneity variables. (DOCX) [file pone.0150111.s001.docx]

**S1 Table:** Variables coordinates on axis of (A) the initial composition variables with the three selected axes of the CA of the composition, (B) the initial configuration variables with the seven selected axes of the PCA of the configuration (C) the initial configuration variables with the seven selected axes of the PCA of the configuration when configuration variables include heterogeneity variables. In dark are highlighted the variables positively correlated to the axis, and in grey the variables negatively correlated to the axes. Only correlations over 0.4 are colored.

A)

|  | Comp 1 | Comp 2 | Comp 3 |
| --- | --- | --- | --- |
| Farmlands | 0.53 | -0.16 | -0.02 |
| Urban | -0.26 | 1.63 | 0.55 |
| Forest | -1.15 | -0.44 | 0.1 |
| Open natural | -0.67 | 0.51 | -0.63 |
| Transport | -0.26 | 1.05 | 0.06 |
| Wetlands/Water | -0.76 | 1.61 | -3.53 |
| Cumulated variance | 41.57 | 70.87 | 86.21 |

B)

|  |  | Comp1 | Comp2 | Comp3 | Comp4 | Comp5 | Comp6 | Comp7 |
| --- | --- | --- | --- | --- | --- | --- | --- | --- |
| Dominant LUC(s) | Patch number | -0.86 | -0.21 | 0.04 | -0.06 | 0.09 | -0.14 | -0.13 |
|  | Mean distance to the nearest neighbourg | 0.03 | -0.83 | 0.14 | 0.08 | 0.05 | 0.46 | 0.28 |
|  | Mean shape complexity | -0.16 | -0.09 | -0.63 | 0.14 | -0.74 | 0.06 | 0.02 |
|  | Mean perimeter | 0.71 | 0.34 | 0.12 | 0.36 | -0.05 | 0.37 | -0.12 |
| Minority LUCs | Patch number | -0.69 | 0.18 | 0.00 | 0.13 | 0.09 | 0.49 | -0.45 |
|  | Mean distance to the nearest neighbourg | 0.52 | -0.55 | 0.01 | 0.14 | -0.03 | -0.27 | -0.58 |
|  | Mean shape complexity | 0.01 | -0.04 | -0.70 | 0.42 | 0.57 | -0.07 | 0.09 |
|  | Mean perimeter | -0.35 | 0.02 | 0.43 | 0.76 | -0.16 | -0.24 | 0.14 |
|  |  |  |  |  |  |  |  |  |
|  | Cumulated variance | 26.71 | 41.54 | 55.24 | 67.04 | 78.59 | 87.91 | 96.41 |

C)

|  |  | Comp1 | Comp2 | Comp3 | Comp4 | Comp5 | Comp6 | Comp7 |
| --- | --- | --- | --- | --- | --- | --- | --- | --- |
|  | LUC diversity | -0,75 | 0,03 | 0,02 | 0,27 | -0,05 | 0,38 | 0,12 |
|  | Shanon equitability | -0,83 | 0,12 | 0,05 | -0,10 | -0,02 | -0,13 | 0,00 |
| Dominant LUC(s) | Patch number | -0,83 | 0,12 | -0,02 | -0,12 | -0,06 | -0,13 | 0,13 |
|  | Mean distance to the nearest neighbourg | -0,04 | 0,83 | 0,05 | 0,10 | -0,01 | 0,09 | -0,54 |
|  | Mean shape complexity | -0,12 | 0,00 | -0,65 | -0,01 | 0,75 | 0,08 | -0,04 |
|  | Mean perimeter | 0,68 | -0,25 | 0,18 | 0,45 | 0,08 | 0,24 | -0,17 |
| Minority LUCs | Patch number | -0,70 | -0,24 | -0,01 | 0,28 | -0,08 | 0,46 | -0,08 |
|  | Mean distance to the nearest neighbourg | 0,41 | 0,61 | -0,01 | 0,23 | 0,04 | 0,17 | 0,60 |
|  | Mean shape complexity | 0,02 | -0,01 | -0,69 | 0,46 | -0,47 | -0,31 | -0,04 |
|  | Mean perimeter | -0,35 | -0,02 | 0,41 | 0,56 | 0,36 | -0,50 | 0,03 |
|  |  |  |  |  |  |  |  |  |
|  | Cumulated variance | 32,07 | 44,13 | 55,14 | 64,86 | 74,12 | 82,48 | 89,7 |
