# Supplementary material for: A Holistic Landscape Description Reveals That Landscape Configuration Changes More over Time than Composition: Implications for Landscape Ecology Studies
Source: PLoS One. 2016 Mar 9;11(3):e0150111. doi: 10.1371/journal.pone.0150111 (PMC4784918; doi:10.1371/journal.pone.0150111)
Supplement: S1 File — (DOCX) [file pone.0150111.s002.docx]

**S1 File: Ecological processes, spatial patterns and landscape descriptions**

A synthesis of the relationships existing between ecological processes and spatial patterns is necessary here to highlight which are the characteristics of landscape description needed to study biodiversity in context of LUC changes.

Species distribution and their related temporal changes of distribution obeys to three ecological spatial constraints (45,46): (i) the environmental conditions that allow the population growing (providing the necessary resources to life and reproduction of the species), (ii) the interaction with other local species that may affect positively or negatively the focal species fitness and behavior (44) and (iii) the accessibility of the location (or location connectivity), depending on the dispersal ability of the species (47).

One way to integrate the three different constraints linked to spatial characteristics defined above for the different species of the landscape is to provide a holistic (4) and non-species centered description of the landscape heterogeneity describing both landscape composition and configuration (7,8). A holistic description of landscape, describing the composition and configuration of all land covers within user-defined elementary units, allows overcoming some limits linked to habitat/matrix descriptions of landscapes:

1. It is of great importance for defining effective habitats (48,49,8). It thus prevents against the problem of habitat identification, even more important for species with varying abilities and requirements during their life cycles and needing different LUCs (50).
2. It may also be informative to properly integrate connectivity constraints in species distribution. The permeability of a LUC type for a species may indeed vary with its location in the landscape and more specifically with its vicinity to a favorable or an unfavorable land cover (51).
3. It is also a way for partly accounting on biotic interactions driven by the LUCs present in the vicinity (46). For instance, a specific composition and configuration of landscape can favor the presence of a superior competitor of the focal species, preventing the presence of this species in the landscape.
4. Finally, it is adapted to the study of communities, which may be composed of species linked to different habitats, with various ecological requirements.
