# Supplementary material for: A Holistic Landscape Description Reveals That Landscape Configuration Changes More over Time than Composition: Implications for Landscape Ecology Studies
Source: PLoS One. 2016 Mar 9;11(3):e0150111. doi: 10.1371/journal.pone.0150111 (PMC4784918; doi:10.1371/journal.pone.0150111)
Supplement: S2 File — (DOCX) [file pone.0150111.s003.docx]

**S2 File: Description of the data set available in S7 File**

Metadata

The provided data set are on 2 excel sheets, one for the composition landscape variables, the second for the configuration landscape variables. The data are the landscape metrics computed from for the years 1982 and 2003, and from the 83 LUC classes cartography simplified in a 6 LUC cartography. These variables are meant to be used in two separate multivariate analyses, a CA for the composition variables and a PCA for the configuration variables, as explain in the manuscript. The initial land cover data set is now freely available on the IAU website (<http://www.iau-idf.fr/liau-et-vous/cartes-donnees/open-data/donnees.html>).

Variables names

In the 2 sheets, the landscape_ID column refers to the identifier of the landscape unit (each the landscape_ID has 2 different rows, one for each year, and is present in both sheets).

Composition: The names of the data are explicit enough and correspond to the percentage of the 6 LUC in each landscape.

Configuration:

The configuration variables are computed for the dominant (over 20% of the landscape area, referred as “dom” in the variables names in the table) and minority land covers 5under 20% of the landscape area, referred as “min” in the variables names in the table).

- nb_patch: number of pathes of dominant or minority LUC in the landscape
- dist: average distance between dominant or minority LUC in the landscape
- shape: average shape index of dominant and minority LUC patches in the landscape, computed as the perimeter/area
- perimeter: average perimeter of the dominant and minority LUC patches in the landscape.
