# Supplementary material for: A Holistic Landscape Description Reveals That Landscape Configuration Changes More over Time than Composition: Implications for Landscape Ecology Studies
Source: PLoS One. 2016 Mar 9;11(3):e0150111. doi: 10.1371/journal.pone.0150111 (PMC4784918; doi:10.1371/journal.pone.0150111)
Supplement: S4 File — (DOCX) [file pone.0150111.s005.docx]

**S4 File: Simplified method applied on the initial set of landscape metrics**

An alternative method, avoiding the step of the multivariate analyses, was also used directly on the initial set of landscape metrics. These analyses had been realized to show the validity of our results using more conventional landscape variables.

The overall framework used to compare the temporal changes in the composition and configuration between 1982 and 2003 is nearly the same as the one depicted in Figure 2, with the following noticeable differences. As multivariate analyses were not used in this alternative method, the landscape variables of composition and configuration were often strongly correlated (Tables A and B). Moreover, the variables were not standardized by the multivariate analyses process and thus their variations were not comparable. Consequently, it was not possible to use the sum of the standard deviations of the composition metrics and of the configuration metrics to standardize all composition and configuration metrics. As reminder, this process was used to be sure i) to obtain similar spatial variations for composition and configuration in spite of different number of variable and thus be sure to focus on the comparison of temporal variations ii) to count only one time the variation shared by several variables. Alternatively, we simply divided each variable by its standard deviation (also computed on the two years of the study) before to subtract the standardized values of 2003 to the ones of 1982 and to compute the standard deviations of the temporal changes (these two last steps as in the other method). Because of the correlation between the variables and of the difference of variable number, we used the mean of the standard deviations of the temporal changes instead of the sum to compare the temporal variations of the composition and configuration. The method did not solve the two problems but partly avoid cumulative effects which should have result from the use of the sum.

A) Correlations between the six landscape composition metrics used in the correspondence analysis.

B) Correlations between the 10 landscape configuration metrics used in the principal component analysis.
