# Supplementary material for: A Holistic Landscape Description Reveals That Landscape Configuration Changes More over Time than Composition: Implications for Landscape Ecology Studies
Source: PLoS One. 2016 Mar 9;11(3):e0150111. doi: 10.1371/journal.pone.0150111 (PMC4784918; doi:10.1371/journal.pone.0150111)
Supplement: S1 Fig — (DOCX) [file pone.0150111.s006.docx]

**S1 Fig: Results of the simplified method.** Standard deviations of the standardized temporal changes for the composition and configuration metrics and their means (DLUC for dominant LUC, MLUC for minority LUC).

**
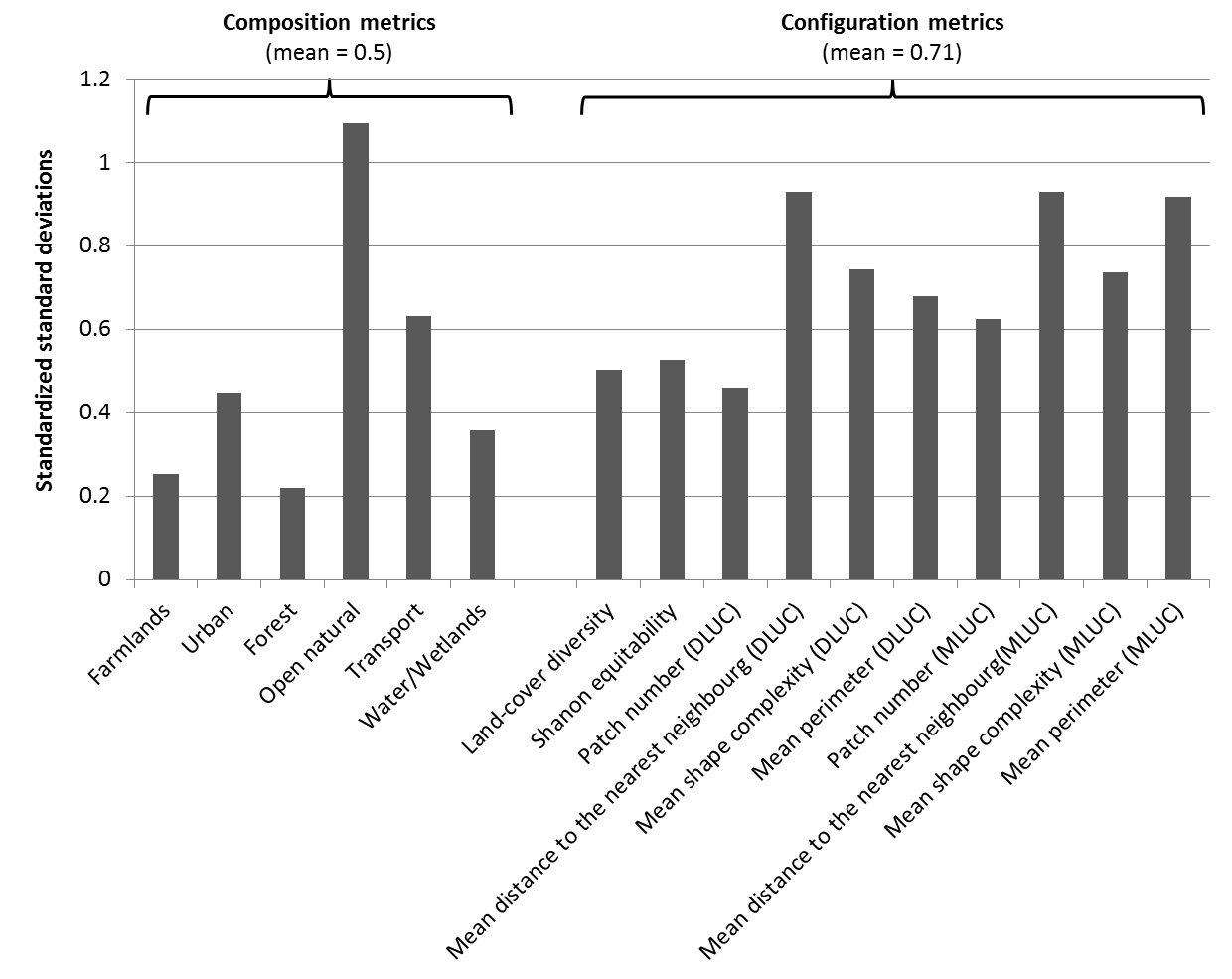
**

The configuration metrics varied in average more over time than did the composition metrics. Because of the correlation between variables and of the absence of standardization for all composition and configuration variables, this method does not allow quantifying the difference of temporal variations between the composition and the configuration.
