# Supplementary material for: A Holistic Landscape Description Reveals That Landscape Configuration Changes More over Time than Composition: Implications for Landscape Ecology Studies
Source: PLoS One. 2016 Mar 9;11(3):e0150111. doi: 10.1371/journal.pone.0150111 (PMC4784918; doi:10.1371/journal.pone.0150111)

**S2 Fig:** Results of the analyses including the heterogeneity variables in the PCA of landscape configuration variables .

1. Averaged temporal variation of the standardized three composition and seven configuration variables from the multivariate analyses for the overall landscapes of the study area between 1982 and 2003 (DLUC for dominant LUC, MLUC for minority LUC).


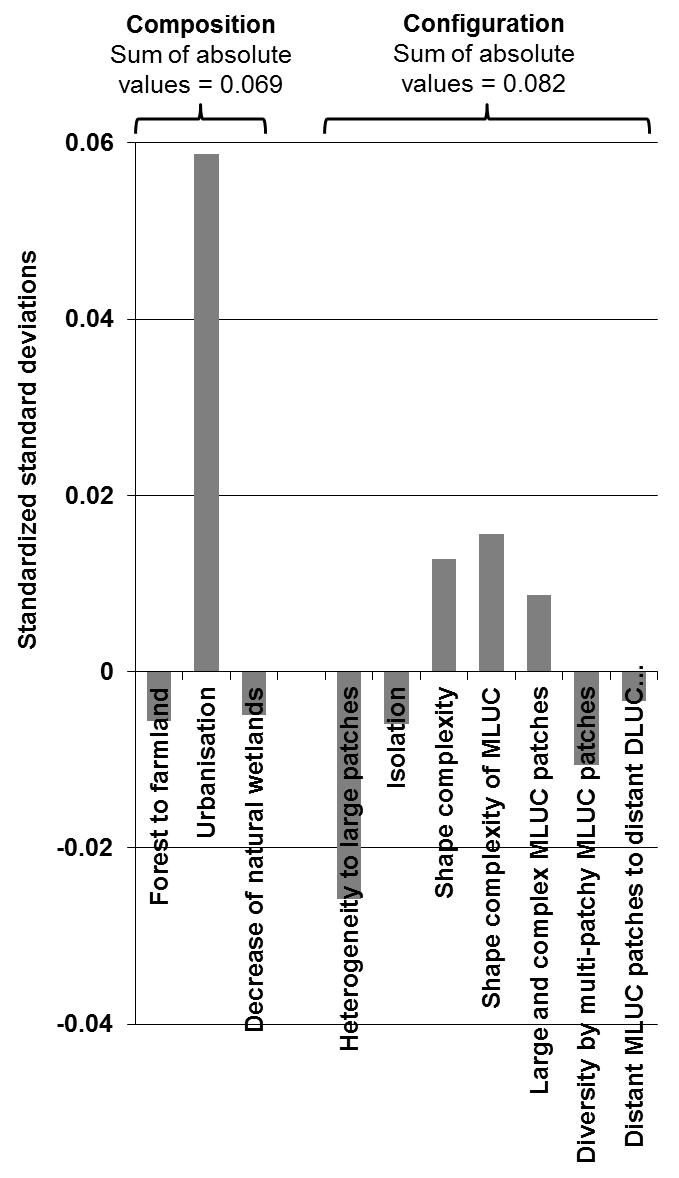


1. Standard deviations of the standardized temporal changes (Chg_LandVar_i_) for the composition and configuration variables and their sum (DLUC for dominant LUC, MLUC for minority LUC).


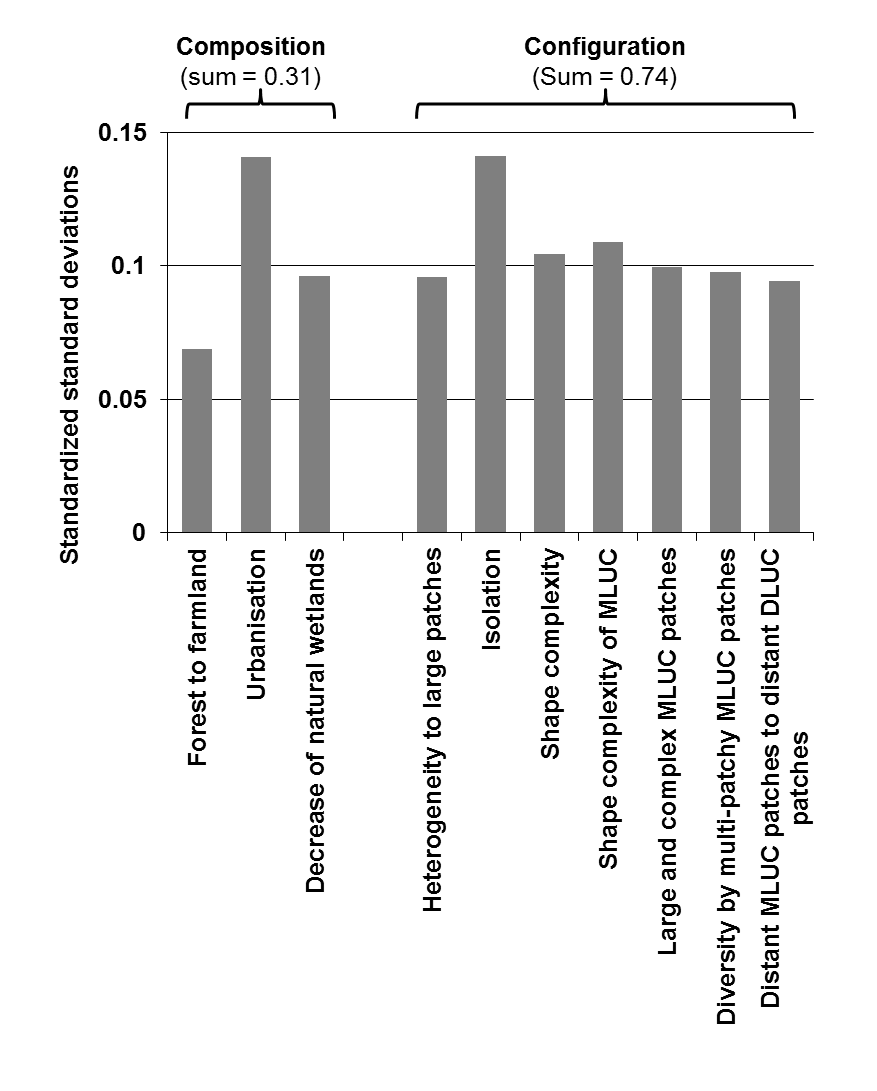

Supplement: S2 Fig — (DOCX) [file pone.0150111.s007.docx]
